# Introducing framework for analyzing non-adherence (FAN)

**DOI:** 10.1186/1745-6215-16-S2-P32

**Published:** 2015-11-16

**Authors:** Ernest Edifor, Sharon Kean, Jane Aziz

**Affiliations:** 1 University of Glasgow, Glasgow, UK

## 

The importance of patient adherence to treatment medication cannot be over emphasised. Some authors have claimed adherence is the secret to a successful clinical trial; intuitively, non-adherence can lead to a trial failure. Non-adherence in clinical trials does not only blur drug efficacy results but could also have huge financial implications; research efforts are incessantly being made to find a “cure” to it.

Fault Tree Analysis (FTA) is a simple way of determining how the combination of basic faults of a system can lead to a total system failure. Initially, it was used in electrical, mechanical, computer and other engineering fields to determine how combinations of components of a system can cause a total system failure. Due to its usefulness, it has been employed in non-engineering fields.

In this work, we introduce the Framework for Analyzing Non-adherence (FAN) and demonstrate how it can improve adherence. FAN is an offline analysis platform which harnesses the strengths of FTA in identifying potential causes of non-adherence and providing valuable information on how to improve the structural design of a clinical trial with adherence in mind.

A software implementation of FAN, called the FAN Tool, is under development. Based on non-adherence information supplied by users, the FAN Tool will allow them to identify rated areas of non-adherence. This will give investigators useful information on where to invest their resources to boost adherence, thereby improving their overall trial success.

